# Cold Atmospheric Plasma Treatment for Pancreatic Cancer–The Importance of Pancreatic Stellate Cells

**DOI:** 10.3390/cancers12102782

**Published:** 2020-09-28

**Authors:** Ruben Verloy, Angela Privat-Maldonado, Evelien Smits, Annemie Bogaerts

**Affiliations:** 1 Plasma Lab for Applications in Sustainability and Medicine-ANTwerp, University of Antwerp, 2610 Wilrijk, Belgium; annemie.bogaerts@uantwerpen.be; 2 Center for Oncological Research, University of Antwerp, 2610 Wilrijk, Belgium; evelien.smits@uza.be

**Keywords:** pancreatic cancer, pancreatic ductal adenocarcinoma, pancreatic stellate cells, cold atmospheric plasma, tumor microenvironment

## Abstract

**Simple Summary:**

This review aims to highlight the potential of cold plasma, the fourth state of matter, as anti-cancer treatment for pancreatic cancer, and the importance of pancreatic stellate cells in the response to this treatment. Currently, a significant lack of basic research on cold plasma considering both pancreatic cancer and stellate cells exists. However, co-cultures of these populations can be advantageous, as they resemble the cell-to-cell interactions occurring in a tumor in response to therapy. Even more, these studies should be performed prior to clinical trials of cold plasma to avoid unforeseen responses to treatment. This review article provides a framework for future research of cold plasma therapies for pancreatic cancer, considering the critical role of pancreatic stellate cells in the disease and treatment outcome.

**Abstract:**

Pancreatic ductal adenocarcinoma (PDAC) is a lethal disease with low five-year survival rates of 8% by conventional treatment methods, e.g., chemotherapy, radiotherapy, and surgery. PDAC shows high resistance towards chemo- and radiotherapy and only 15–20% of all patients can have surgery. This disease is predicted to become the third global leading cause of cancer death due to its significant rise in incidence. Therefore, the development of an alternative or combinational method is necessary to improve current approaches. Cold atmospheric plasma (CAP) treatments could offer multiple advantages to this emerging situation. The plasma-derived reactive species can induce oxidative damage and a cascade of intracellular signaling pathways, which could lead to cell death. Previous reports have shown that CAP treatment also influences cells in the tumor microenvironment, such as the pancreatic stellate cells (PSCs). These PSCs, when activated, play a crucial role in the propagation, growth and survival of PDAC tumors. However, the effect of CAP on PSCs is not yet fully understood. This review focuses on the application of CAP for PDAC treatment and the importance of PSCs in the response to treatment.

## 1. Introduction

To date, cancer remains as a highly complex group of diseases characterized by the disrupted metabolic activity, altered repair mechanisms, and redundant signaling pathways across various cell types [1]. In addition, the interaction of cancer cells with other cells in the tumor microenvironment (TME) can determine the treatment outcome. This dynamic nature of cancer can favor drug resistance, which represents a challenge for cancer treatment [2]. Cancer research is currently directed to develop new therapeutic approaches that can efficiently disrupt cancer hallmark features and overcome the limitations of current treatments. Cold atmospheric plasma (CAP), a new tool from the field of physics, has shown great potential for its therapeutic capabilities against cancer. CAP has shown to effectively eliminate several cancer cell types both in vitro and in vivo [3,4,5,6]. Even more, the first clinical pilot studies in head and neck cancer patients have shown a positive result, reducing the microbial load in oral carcinoma lesions [7,8]. The advantage of CAP is its multimodal nature that can simultaneously attack multiple targets in cancer cells, overcoming some of the limitations of current therapies.

Hard-to-kill cancers have highly developed properties that cause invasiveness, metastasis, and resistance towards therapy, among others. One of these aggressive cancers is pancreatic cancer, which is predicted to become the third global leading cause of death by cancer in the near future [9]. In 2018, global pancreatic cancer incidence and mortality were 458,918 and 432,242, respectively [9]. Pancreatic ductal adenocarcinoma (PDAC), the most common type of pancreatic cancer with approximately 85% of all cases [10], is characterized by early metastasis and a desmoplastic reaction. The formed dense, fibrous tissue acts as a resilient shield towards chemotherapy and radiotherapy. The stromal pancreatic stellate cells (PSCs) significantly contribute to the hallmarks of PDAC and play a key role in creating an ideal TME for the survivability, progression and resilience of the tumor. CAP treatment could serve as a combinational therapy to improve current treatment of this aggressive cancer. It is known that CAP treatment can alter the extracellular matrix (ECM) and enhance the delivery of therapeutics drugs [11], besides eliminating cancer cells. Therefore, the combination of these therapeutic strategies could lead to a potentially synergistic effect and provide an improvement for PDAC treatment.

In this review, we discuss the background, state-of-the-art and future of CAP therapy for PDAC, with special attention to its effect on PSCs and the TME, and the potential benefits of the combination of CAP with chemotherapeutic strategies. CAP treatment for PDAC is yet in a fundamental stage of research in which no clinical results have yet been obtained. As CAP research progresses towards the development of future therapies, it is important to consider the role of other cell types in the TME of PDAC, such as the PSCs, prior to considering clinical trials. Further studies of the complex interactions of cells in the TME of PDAC in vitro and in vivo will help to more accurately predict CAP treatment outcomes.

## 2. Plasma and Its Medicinal Properties

### 2.1. What Is Plasma?

Plasma, the fourth aggregate state, is a (partially) ionized gas, which includes electrons, various types of ions, radicals, and excited species, besides the gas molecules. Plasmas can be generated by coupling sufficient quantities of energy to a gas to induce ionization. During this process, the atoms or molecules lose one or several electrons, resulting in the generation of a mixture of neutral, excited and charged species that exhibit collective behavior [12]. The excited species emit photons when decaying to the ground state (or a lower level), which explains the glow emitted by plasma. The neutral species and ions are commonly referred as “heavy” species, while the electron and photons as “light” due to discrepancies in their mass.

Natural and man-made plasmas are generated under a wide range of conditions, including low, atmospheric and high pressures, electron temperatures ranging from 10^2^ to 10^8^ K and electron densities ranging from 10^3^ to 10^33^ electrons/m^3^, respectively [13]. In plasmas, the temperature is determined by the energies of the heavy particles and their degrees of freedom (translational, rotational, vibrational and the ones related to electronic excitation) [13]. Plasmas can be broadly classified into two categories: thermal and non-thermal plasmas. While heavy and light particles in thermal plasmas approach local thermodynamic equilibrium at temperatures up to 100,000 K and more (as in fusion reactors), non-thermal plasmas are not in thermodynamic equilibrium. Instead, the high temperature of the electrons determines the ionization and chemical processes, but the low temperature of heavy particles determines the macroscopic temperature of plasma [13,14].

Non-thermal plasmas generated at atmospheric pressure, commonly referred to as cold atmospheric (pressure) plasmas (CAPs), are particularly important in biomedicine. For example, man-made CAPs currently used in medicine include plasma blades for coblation, cauterization and thermocoagulation [14]. All of these operate at temperatures of 70–90 °C and part of the effect is due to heat transfer [14]. Another type of CAPs includes those operating at temperatures below 40 °C, where its effect is predominantly due to the reactive species, ions, and electrons, and to a lesser degree, heat, photons, and electromagnetic fields [15,16,17].

CAP has received increasing attention for oncological research in the last decade, as it generates a rich cocktail of reactive oxygen and nitrogen species (RONS), which are able to induce cellular responses that could lead to cell death, with certain selectivity to cancer cells [17,18,19]. The configuration of the most common CAP sources used for cancer research is described in the following section.

### 2.2. Plasma Devices

**Figure 1:**
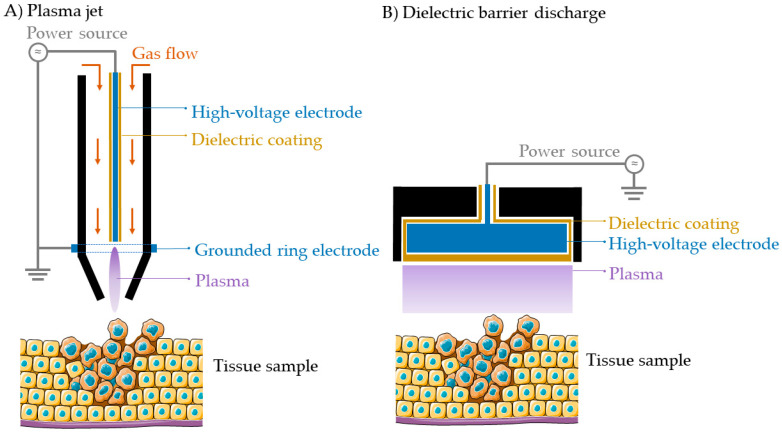
Schematic representation of the two most common plasma devices for oncological purposes. (**A**) Plasma jet, (**B**) Dielectric barrier discharge (DBD).

The most common types of CAP sources used in cancer research are the plasma jet (Figure 1A) and the dielectric barrier discharge (DBD) (Figure 1B). The plasma jet operates using an active gas flow (e.g., a noble gas, such as helium or argon, or ambient air) while the DBD can operate in air without an active gas flow. Plasma is formed upon the ionization of gas between the powered and ground electrode by the application of a high voltage, with (typically) one of the electrodes coated with a dielectric material, e.g., quartz [20]. In case of the plasma jet, the plasma plume extends outside of the device towards the target sample for treatment. In case of the DBD, the target tissue or material acts as the second electrode, and plasma is formed between the device electrode and the target tissue or material. The amount and type of RONS produced in a CAP depends on the configuration of the plasma device, the frequency, and power, as well as the distance between the nozzle and the target sample, the treatment time and the gas used for its generation, making it a versatile tool that can be modulated to improve the treatment outcome [16]. Biomedical CAPs can be applied directly or indirectly to the target tissue [17]. In direct treatments, CAP is brought into close contact with the target tissue, which benefits from the exposure to both short-lived and long-lived species produced by CAP. In indirect treatments, CAP is applied to a solution (phosphate buffered saline, water, saline solution, etc.) where CAP-derived RONS (long-lived reactive species) are stored in the liquid for their future application on the target tissue.

### 2.3. Cold Plasma for Medicine–Important Reactive Species and Their Effects on Cells

For biomedical applications, CAPs operating at temperatures below 40 °C are commonly used [21], as this temperature is below the tissue damage threshold of 43 °C [22]. CAPs generate a variety of RONS with biological importance, such as superoxide (O_2_^−^•), hydrogen peroxide (H_2_O_2_), hydroxyl radical (•OH), singlet oxygen (^1^O_2_), ozone (O_3_), organic radicals (RO• and RO_2_•), nitric oxide (•NO), nitrogen dioxide (•NO_2_) and peroxynitrite (ONOO^−^). The amount and type of generated RONS depends on the treatment conditions, as mentioned before. Biomedical CAPs have proven to be effective against a variety of microorganisms, including multi-drug resistant pathogens, which is useful for e.g., sterilization of living tissue and non-living objects [23]. However, we will only focus on the application of CAP treatment for oncological purposes.

Oxygen is a molecule of dual character towards organisms. It is a key factor to sustain life, but could also induce harmful oxidative effects to biomolecules. Cells are equipped with anti-oxidants to maintain homeostasis [16], but a misbalance in pro-oxidants and anti-oxidants can cause oxidative stress on cells [16,24]. Under normal conditions, intracellular levels of RONS are maintained low by the action of enzymes, e.g., superoxide dismutase and catalase [24,25], and non-enzymes, e.g., vitamin C, E and β-carotene [24]. In cancerous cells, intracellular levels of RONS are higher due to an increased metabolic activity (also known as the Warburg effect) [16]. During CAP treatment, extracellular RONS are applied to the tissue, having a strong influence on the cellular response. The consequences of the direct and indirect effects of these exogenous CAP-derived RONS on cells involve complex mechanisms of action that are not yet fully understood. However, some knowledge is already available on how CAP-derived RONS affect the cell structure and metabolism [16,26,27]. NADPH oxidase-1 is a membrane-associated protein and expresses membrane-bound catalase and superoxide dismutase. Singlet oxygen can inactivate catalase, causing the generation of secondary singlet oxygen, which can lead to apoptosis [26,27]. Another contributing mechanism is through the highly reactive hydroxyl radical (•OH), as it causes lipid peroxidation and DNA damage, which leads to apoptosis [16,27]. Aquaporins also play a role because they allow hydrogen peroxide to enter the cell [28,29], which can induce cell death by oxidative DNA damage, such as DNA strand breakages, and covalent cross-linking of DNA and proteins [30]. Intracellular signaling pathways also provide an opportunity for cancer cells to manipulate tumor growth and survival, with the PI3K/Akt and RAS/MAPK pathways being the most commonly studied pathways. It has been shown that CAP can induce apoptosis via these pathways, as demonstrated for glioblastoma cells [31], and could also be an interesting mechanism against PDAC [32]. 

There exists no clear definition yet for the dose of plasma-induced RONS [33], however, it is known that the amount of RONS can have cell proliferative or cytotoxic effects [34]. At low concentrations of RONS (mild conditions), CAP can have positive effects, e.g., promote wound healing by blood coagulation, cell proliferation, angiogenesis and tissue regeneration [23,35]. At higher concentrations of RONS (harsh conditions), CAP can induce cell death, as the oxidative balance in cancer cells and tissues is disturbed, which is an important feature for the elimination of cancer cells. This biphasic response is called hormesis, an adaptive response on disturbance to maintain homeostasis [36]. This lies at the basis of the application of plasma medicine, as mild treatments are used for wound healing, while harsh treatments are effective against cancer cells. This can be concluded from the different induced cellular responses by CAP-derived RONS: lipid peroxidation [16], destruction of lipid membranes [37], endogenous RONS generation [16], DNA damage [17,38], reduced proliferation [39], cell cycle arrest [34,40], cell detachment [21], decreased cell migration [21], apoptosis [21,34,40,41], non-apoptotic cell death [4], e.g., necrosis [21], immunogenic cell death [40,42], and an increased sensitivity to chemo- [43] and radiotherapy [44]. 

CAP treatment as an anti-cancer therapy has proven effective against multiple cancer types, e.g., lung [45,46], head and neck [4,47], breast [48,49], melanoma [5,50], and colorectal [51,52] and pancreatic cancer [42,53]. In oncology, CAP has also shown to favor the activation of the immune system, e.g., macrophages and the production of damage-associated molecular patterns to eliminate cancer cells [54]. Because of the different possible effects, the treatment conditions are important and determine the level of damage inflicted to the tissue. CAP treatment offers great potential as a combinational therapy due to its effect on biological cells, especially for cancer types where conventional therapies fail to improve the survival rates, which is the case for PDAC. 

### 2.4. Is There a Selectivity towards Cancer Cells?

Research suggested that cancer cells are more sensitive towards CAP treatment than healthy cells [4,48,55,56,57,58], providing a selective effect. The probable underlying reason is the increased concentration of RONS present in cancer cells, as discussed before [15,16,59]. The RONS levels in cancer cells are higher than in healthy cells and therefore closer to the oxidative damage threshold value [60]. Another factor could be their higher vulnerability to DNA damage due to an abnormal dividing capacity [61]. The high expression of aquaporins [62,63,64] and a lower level of cholesterol in the plasma membrane in cancer cells [65,66] could also increase the transport of RONS into the cytoplasm, damaging cancer cells more than their healthy counterpart. However, the above observations on selectivity must be evaluated critically. Recent research has provided evidence on the importance of the treatment conditions, such as the difference between the cell culture media used for different cell lines and the cell type of healthy and cancerous cells [19], and it is recommended that the same treatment conditions must be applied to compare the effect on both cancer and healthy cells. During in vivo treatment, cancerous and normal cells are treated simultaneously and without difference in tissue environment (cf. culture medium). However, the microenvironment needs to be resembled during in vitro experiments as much as possible to be able to predict the treatment response in vivo and to reduce the chances of failure of future CAP treatments in patients.

## 3. Pancreatic Ductal Adenocarcinoma (PDAC)

Pancreatic ductal adenocarcinoma (PDAC) is one of the most lethal types of cancer and is refractory to most of the current treatment approaches, as the five-year survival rate of patients subjected to surgery, radio- and chemotherapy are barely 8% [67]. The improvement of treatment strategies for PDAC has increased the five-year survival rates only from 6% to 8% over the last few years [68,69]. These numbers highlight the need for therapeutic alternatives for this disease. High contributing factors to the development of PDAC include obesity, smoking and a family history of the disease [70]. The acinar (exocrine) and ductal (epithelial) cells of the pancreas are important in this development [67]. Upon environmental stimuli, e.g., stress, inflammatory conditions, etc., acinar cells transform into ductal-like cells [71]. Through this transformation, the acinar cells become more sensitive towards mutations in, e.g., proto-oncogene KRAS, resulting in pancreatic epithelial neoplasia [67,71]. Followed by other oncogenic mutations, these can lead to the progression towards PDAC.

PDAC is characterized by the development of early metastasis and desmoplasia, the formation of a dense fibrous tissue. This tissue accounts for 50–80% of the total tumor volume and is largely a consequence of the activity of stromal cells present in PDAC [72]. In PDAC, the TME is responsible for the aggressiveness, resistance to therapy, rapid growth, invasiveness, and survival in hypoxic and low-nutrient environments, due to a high interstitial pressure in PDAC.

### 3.1. Current Therapies for PDAC

Currently, surgery with adjuvant chemotherapy is the only potential treatment for PDAC [73]. However, only 15–20% of patients have a chance of successful surgical resection at time of diagnosis, and it is only performed when a positive response to neoadjuvant therapy is observed [74,75]. Surgical resection is not an option when the tumor is too advanced or metastases have already been formed [67,75]. Thus, this technique presents some disadvantages for the treatment of PDAC: (i) it cannot remove metastases; and (ii) remaining cancer cells in the tumor site have the risk to form secondary tumors. In addition, bleeding, pain, damage to adjacent tissues or organs have been reported as negative side effects. The combination of surgery with other therapies is still highly recommended for circulating cancer cells or metastatic niches, as well as to avoid recurrence of the tumor.

The most commonly used chemotherapeutic drugs for patients with metastatic, locally advanced, or unresectable PDAC are FOLFIRINOX (FFX) and gemcitabine combined with nab-paclitaxel (GNP) [67]. FFX was observed to be more effective than GNP, with overall survival of approximately 14 months compared to nine months, respectively [76]. In another study, the overall survival of patients treated with FFX was 11.1 months, while it was only 6.8 months with gemcitabine alone [77]. The enhanced effect of combining gemcitabine with nab-paclitaxel has been observed, by an overall survival of 8.5 months for GNP and only 6.7 months for gemcitabine alone [78]. Although an increase in overall survival is observed, this increase is only with a few months. The high toxicity of chemotherapeutics in healthy cells is particularly important, as it prevents their use in the elderly and patients with poor health status [67]. 

Compared to other types of cancers, PDAC has an increased resistance to radiotherapy due to the desmoplastic shield [79]. Radiotherapy is used for improving surgical possibilities or together with chemotherapy, known as radiochemotherapy [80]. Radiosensitizing agents, such as capecitabine, gemcitabine and fluorouracil (5-FU), combined with radiotherapy can reduce the radioresistance of PDAC [67]. Adjuvant radiotherapy treatment has great potential to attack both cancer cells remaining in the tumor bed after surgery and the circulating, invasive cells. Nevertheless, 71–76% of patients relapsed within two years [73,80]. Radiochemotherapy as neoadjuvant treatment could offer the advantage of improving surgical possibilities for PDAC patients with borderline-resectable tumors, yet more research on this strategy is necessary [73,80]. 

Immunotherapy was stated to be the biggest breakthrough in cancer research in 2013 [81] and is also actively used in research for PDAC [82], but to date, its efficacy in PDAC is still limited [83]. More efforts are needed in this direction to overcome the immunosuppressive and therapy-resistant TME of PDAC [84,85] and to identify specific target molecules on cancer cells, without targeting normal cells [86].

As discussed here, the current conventional therapies cannot significantly improve the survival of PDAC patients. Novel combination therapies for PDAC could be used to improve the therapeutic outcome. The ability of CAP to eliminate cancer cells, with certain degree of selectivity towards unhealthy cells, could be explored in combination with chemotherapy to treat this lethal disease [16]. In the future, CAP treatment could be considered as a new treatment strategy for PDAC, in addition to the existing ones [87].

### 3.2. The PDAC Tumor Microenvironment–Importance of PSCs

**Figure 2:**
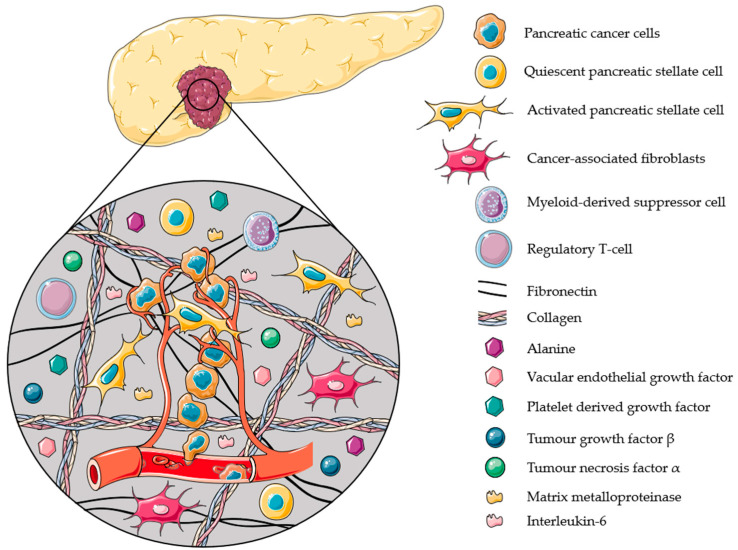
Schematic representation of the TME of PDAC and its dense desmoplastic stroma. Pancreatic stellate cells (PSCs), as main type of cancer-associated fibroblasts in this case, account for nearly 50% of the total stroma and are typically activated in PDAC. PSCs recruit immunosuppressive cells (e.g., MDSC and T-reg) and secrete extracellular matrix molecules (e.g., collagen and fibronectin), immunosuppressive (e.g., TGF-β) and inflammatory (e.g., IL-6 and TNF-α) cytokines, pro-angiogenic factors (e.g., VEGF), matrix metalloproteinases (e.g., MMP-2 and MMP-9), growth factors (e.g., PDGF), and non-essential amino acids (e.g., alanine). The complexity of this TME due to PSCs causes PDAC to acquire crucial properties like rapid growth, high invasion and metastatic potential, survival in hypoxic and low-nutrient conditions, and resistance to therapy.

PSCs are the major contributors in the creation of the ideal TME of PDAC (Figure 2). They are responsible for the secretion of matrix metalloproteinases (MMPs), which degrade and remodel the ECM. This ECM remodeling has several effects: (1) it allows growth factors, e.g., platelet-derived growth factor (PDGF), to be released, causing tumor growth; (2) it increases pancreatic cancer cell (PCC) motility to promote invasion and metastasis; (3) it increases intra-tumoral pressure and hypoxia, and (4) it creates a barrier and provides resistance towards chemotherapy [79,88]. Besides creating an ideal TME for tumor growth and resistance towards treatments, PSCs are also responsible for inducing an inflammatory response and creating an immunosuppressive environment. PSCs recruit immunosuppressive cells, such as myeloid-derived suppressor cells (MDSC) and regulatory T-cells (T-reg) by secreting C-X-C motif chemokine 12 (CXCL12) [88], inflammatory cytokines, e.g., interleukin-6 (IL-6) [89], and immunosuppressive cytokines, e.g., transforming growth factor β (TGF-β) [88]. In the low-nutrient and hypoxic environment of PDAC tumors [90,91], PSCs can contribute to the PDAC metabolism by secreting non-essential amino acids (e.g., alanine and aspartate), which can be used as an alternative energy source during a scarcity of glucose. The secretion of these amino acids results from an autophagic PSC death, induced by the PCCs [92]. In these conditions, PCCs with defects in their apoptotic mechanism can survive and overtake normal cells. It has also been reported that PSCs express pro-angiogenic factors, e.g., vascular endothelial growth factor (VEGF) receptors, providing a pathway for metastasis [93].

### 3.3. Activated PSCs: Key Players for the Progression of PDAC

**Figure 3:**
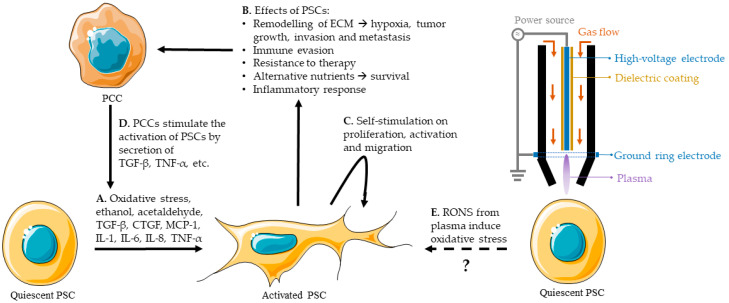
Mechanism of reciprocal stimulation of PSCs and PCCs and the hypothetical influence of CAP (represented by treatment with a plasma jet). (**A**). Activation of PSCs due to different stimuli, e.g., oxidative stress, ethanol, acetaldehyde, TGF-β, CTGF, MCP-1, IL-1, IL-6, IL-8, TNF-α. (**B**). PSCs secrete immunosuppressive and inflammatory cytokines, matrix metalloproteinases, growth factors, pro-angiogenic factors, ECM molecules, and non-essential amino acids, by autophagic cell death and they recruit immune cells. These create an environment that allows PCCs to proliferate, evade cell death and the immune system, survive in hypoxia and low-nutrient environments, recruit immune cells and resist therapy. This has a major stimulation effect on PCCs and the survival and progression of the tumor. (**C**). Self-stimulation of PSCs on their proliferation, activation and migration. (**D**). Stimulation of the activation of PSCs by PCCs. (**E**). Unknown effect of CAP-derived RONS on PSC activation.

Quiescent PSCs are responsible for maintaining the stromal composition in the healthy pancreas, and are involved in the storage of vitamin-A rich lipid droplets [79,88]. When activated, the quiescent PSC changes from a regular, circular phenotype to a star-shaped, myofibroblast-like phenotype. This activation is the result of different stimuli, such as TGF-β, tumor necrosis factor (TNF-α), oxidative stress, etc., and is typical for PDAC (Figure 3A) [79,94]. 

One of the main reasons for the resistance of PDAC to treatment is the desmoplastic reaction induced by activated PSCs [79]. This reaction causes high intra-tumoral pressure and acts as a shield that surrounds the tumor, limits the blood flow and the delivery of therapeutic agents, oxygen, and immune cells [90]. Activated PSCs are key players in PDAC and can induce tumor growth, PCC and PSC motility, invasion and metastasis, immune evasion, an inflammatory response, hypoxia, a metabolic alternative, etc. (Figure 3B), as discussed before. The secretion of cytokines and other molecules by PSCs, discussed in Section 3.2, will also cause a proliferative and migrational effect on PSCs, and sustain their activation (Figure 3C). In addition, PCCs stimulate PSC activation and growth, resulting in a reciprocal stimulation (Figure 3D). This loop of stimulation is a major reason for the aggressiveness of PDAC [79]. 

It is known that oxidative stress can induce the activation of PSCs [79,88,94]. CAP treatment provides an external source of RONS that, in principle, could favor the activation of PSCs or exacerbate their response to RONS (Figure 3E). 

Activated PSCs can be distinguished from quiescent ones by evaluating the expression of specific markers. Commonly used markers for activated PSCs in literature are α-SMA, GFAP, desmin, vimentin, and FAP (Table 1). Besides these markers, epithelial-to-mesenchymal transition (EMT) markers could also be useful, as the EMT-transition process is suspected to be related to the activation of PSCs [95]. Some of these markers and relevant to investigate PSCs are e-cadherin, n-cadherin, and slug. 

Except for α-SMA, there is still uncertainty on the expression profiles of the markers for quiescent and activated PSCs. Tian et al. [95] have compared the expression of e-cadherin, n-cadherin, vimentin and slug between quiescent and activated PSCs, validating the expression profile given in Table 1. However, the authors stated that PSCs upregulate the expression of α-SMA, vimentin, desmin, and GFAP, while they do not make a distinction between markers for quiescent and activated PSCs. In previous publications, these markers are suggested to be related to PSC activation, but no comparison of the protein expression between quiescent and activated PSCs was made nor did the cited literature experimentally validate these differences in expression [79,96,97,107,108]. Based on current research, it is difficult to determine the exact difference in expression profiles between quiescent and activated PSCs for these markers (Table 1). Therefore, a pool of markers is still required to evaluate the activation of PSCs, as was partly done by Tian et al. [95].

### 3.4. PSCs as Major Contributors to the Hallmarks of PDAC 

The aggressiveness of the disease can be resumed in a set of hallmarks for PDAC that explain and reveal the importance of PSCs in the PDAC TME [67,79,109], as PSCs can:

- Increase resistance to cell death by apoptosis in hypoxic regions
- Favor an immunosuppressive and inflammatory TME
- Induce a desmoplastic environment that shields the tumor and increases resistance to therapy
- Induce angiogenesis, favoring invasion and metastasis
- Provide an adaptive metabolic strategy when glucose is scarce
- Boost an excessive proliferative capacity and reciprocal stimulation due to the cross-talk between PCCs and PSCs

It is clear that the stromal PSCs significantly contribute to the hallmarks and act as the guardians of PDAC. They favor the rapid growth of PCCs, their survival in abnormal conditions, resistance to therapy, etc., by creating the ideal TME. This cross-talk between PSCs and PCCs is an important aspect to tackle and is key for the development of novel therapeutic approaches for PDAC.

## 4. State-of-the-Art on CAP Treatment of PDAC

In this section, we discuss all the current research on CAP treatment of PDAC to the best of our knowledge. Studies that only included PCCs will be described first, followed by the limited number of reports that also investigated PSCs. It is due to this limited number of publications including PSCs in their research that we want to emphasize the importance of these cells in the response to treatment for future CAP research. These studies are summarized in Table 2.

### 4.1. CAP Treatment of PCCs

Multiple efforts have been made to determine the mechanisms of action and efficacy of CAP on PDAC cells. One of the first studies on CAP treatment for PDAC investigated the surface temperature of cell culture dishes (to evaluate the safety of this treatment method) and cell death (determined by an Annexin-V-FITC/DAPI assay) of in vitro tumors for murine PDAC 6606PDA cells with a plasma jet [110]. Results revealed an increase in surface temperature of only approximately 2–3 °C after 5–20 s and a significant increase in cell death after CAP treatment compared to controls.

Brullé et al. used a plasma jet to investigate the combinatorial effect of CAP treatment and gemcitabine on tumor growth by tumor volume, weight and bioluminescence (expression of luciferase in Mia Paca-2 cells) [43]. Both gemcitabine and CAP treatment caused significant tumor growth reduction, but CAP treatment produced a larger anti-tumor effect. The combination of CAP and gemcitabine enhanced the anti-tumor effect by 33% compared to only CAP treatment.

Bekeschus and Freund et al. have demonstrated that direct CAP treatment with a plasma jet significantly decreased the metabolic activity of Mia PaCa-2 and PANC-1 in 2D cultures in vitro, which correlated with a reduced cell viability [111]. PANC-1 showed higher resilience against CAP treatment (−6% metabolic activity) compared to Mia PaCa-2 (−38% metabolic activity). In addition, the authors performed similar assays using the in ovo model. Both cell lines showed a reduction in the metabolic activity in tissues collected from this experimental model, but PANC-1 showed a higher sensitivity to CAP treatment. Further experiments in 3D spheroids in vitro suggest that CAP does not have a stimulating effect on the metastatic potential of PDAC cells [111].

Another study investigated the effect of indirect CAP treatment with a plasma jet on apoptosis (determined by caspase 3/7 detection) and cell viability (determined by a WST-1 proliferation assay) of the human PDAC cell lines BxPC-3, Mia PaCa-2, Capan-2, and PANC-1 [112]. CAP treatment of 1 min on the culture medium resulted in differences in sensitivity between cell lines. Capan-2 showed higher cell death than other cell lines for all three different seeding densities and PANC-1 did only show significant cell death for the lowest seeding density, which is unlike other cell lines. The higher resilience of PANC-1 to CAP treatment compared to Mia PaCa-2 was also seen in this study, but only to a minor extent compared to previously mentioned study of Bekeschus and Freud et al. Changing treatment time to 3 or 5 min resulted in effective cell death for all cell lines with a slightly higher cytotoxic effect after 5 min of treatment. Interestingly, 5 min of CAP treatment showed highest cell death for the highest seeding density in all cell lines, which was not observed for the other treatment times. Comparison of the cell viability between Capan-2 cancer cells and human pancreatic epithelial non-cancer cells (HDPE6/C7) showed a higher selectivity to CAP treatment for the cancer cells. Besides these findings, apoptosis was suggested to be induced due to the observation of caspase-3/7 activation and typical morphological changes. The anti-tumor effect of CAP treatment was validated in mice by a reduction in tumor volume of 64% compared to the control group.

Azzariti et al. investigated the effect of DBD plasma-activated medium on PANC-1 cells, assessing cell viability (by colorimetric proliferation assay) and cell death (by Annexin-V-FITC apoptosis assay) [113]. CAP treatment significantly reduced cell viability and increased apoptosis in PANC-1 cells, however, autophagy was not observed. Based on the exposure of calreticulin and release of adenosine triphosphate, it was suggested that CAP induced immunogenic cell death (ICD), a process that activates the immune system to promote cell death in cancer cells and evoke a long-lasting protective antitumor immunity [115].

Chen et al. investigated the effects of plasma-activated saline solution with a plasma jet on the survival of BxPC-3 (human PDAC cell line) and H6c7 (human pancreatic epithelial non-cancer cell line) cells [53]. A cell viability assay based on relative metabolic activity compared to untreated controls has shown a higher sensitivity of BxPC-3 cells to indirect CAP treatment.

Another study with a plasma jet investigated the murine PDAC cell line 6606PDA [6]. In vitro, cell metabolic activity (determined by Resazurin degradation) and cell proliferation (determined by bromodeoxyuridine incorporation) both decreased after CAP treatment, with a larger effect for 6606PDA cells compared to fibroblasts. No difference between indirect and direct treatment was seen. In vivo, cell apoptosis (determined by TUNEL staining) was significantly increased after indirect CAP treatment compared to controls. Apoptosis was also observed in deeper parts of the tumor, implicating penetration of the medium. No side effects were observed from these CAP treatments on mice.

### 4.2. CAP Treatment of PCCs and PSCs

To date, although PSCs play a key role in the treatment outcome of PDAC, only two studies, both from our group, have addressed the effect of CAP on these cells [42,114].

We investigated the potential of CAP treatment to induce ICD with plasma-activated phosphate-buffered saline (pPBS) using a plasma jet [42]. Four pancreatic cancer cell lines (Mia PaCa-2, PANC-1, BxPC3 and Capan-2) and three PSC lines (hPSC128, hPSC21, and RLT-PSC) were included. Mia PaCa-2 showed the highest percentage of cell death (determined by Annexin V and propidium iodide (PI) flow cytometric staining) after pPBS treatment, followed by PANC-1. PSCs showed less sensitivity to pPBS treatment than PCCs. Different ICD markers (CRT, ATP, HMGB1) were investigated and suggested that indeed ICD is induced in the PCCs, and not in PSCs.

In the second study, indirect CAP treatment with a plasma jet was used for Mia PaCa-2 and BxPC3 cancer cells and the PSC cell line hPSC128-SV with plasma-treated medium and plasma-treated water [114]. Cell viability (determined by MTT assay) was hardly reduced after CAP treatment on all three cell lines. Observed cell death was mostly due to apoptosis and necrosis (determined by Annexin V/PI staining).

To date, there are no studies yet on the effect of CAP on co-cultures of PDAC and PSC cells. The lack of knowledge of this effect could give unforeseen drawbacks when moving onto in vivo PDAC tumors or clinical trials. Therefore, it is important to include PSCs in the experimental approaches of future studies.

## 5. Future Perspective on CAP Treatment for PDAC with Attention to PSCs

An important research question is: how could we use CAP to fight PDAC? As presented above, the current findings on the application of CAP for PDAC in vitro and in vivo show an interesting and promising potential. Yet, it is clear that more studies are needed to determine its effect on the PDAC TME, particularly on the PSCs. This is an important factor that must be considered for the development of new CAP therapies for PDAC. In general, the goal is to reduce the effects of PSCs that create the ideal TME for PDAC. Reprogramming of PSCs is one possibility, but it is also highly interesting to look into the direct influence of CAP treatment on the effects induced by PSCs. Therefore, the ECM decomposition, autophagic cell death, the recruitment of immunosuppressive cells, etc. should be considered as well.

### 5.1. Eliminating or Reprogramming PSCs?

The most important aspect to investigate is whether CAP treatment induces the activation of PSCs or enhances the deleterious activity of the already activated PSCs. This would cause an increased production and secretion of the factors that contribute to the formation of an ideal TME, desmoplastic shield and reciprocal stimulation of PCCs. CAP has the potential to eliminate cancer cells, and PSCs have shown to resist the oxidative damage inflicted by CAP. Reprogramming of PSCs by CAP, rather than depleting them, could be a better strategy against PDAC, as the elimination of PSCs has been observed to increase the aggressiveness of the disease [88]. Therefore, CAP treatment conditions need to be found that affect the PSCs and the TME, and that can eliminate PCCs, without eliminating all PSCs. Tumor PSC reprogramming has been proposed as an alternative strategy to regulate the activity of activated PSCs and to improve the therapeutic outcome [88,116]. One way to do this is by activating the p53 pathway in PSCs, which can revert activated PSCs to their quiescent state and could majorly suppress all the effects of the activated PSCs [116]. CAP could potentially activate the p53 pathway by DNA damage, nitric oxide and other oxidative radicals, serving as a possible tool for PSC reprogramming [117]. An ideal outcome would be that CAP reverts the activation of PSCs to restore the fibrotic stromal homeostasis in PDAC. However, it is currently unknown whether the levels of RONS derived from CAP treatment could perpetuate PSC activation rather than reducing it. This aspect of CAP treatment of PSCs in PDAC tumors requires attention and further study.

### 5.2. Targeting the Extracellular Matrix

Another emerging question is whether CAP is able to damage and weaken the ECM and the dense stroma, therefore weakening the desmoplastic shield. There is a balance between ECM production and degradation to maintain a normal ECM, which is influenced and partially regulated by RONS [118]. RONS are also responsible for matrix-degrading enzymes, such as MMPs, for ECM turnover [118], which could be interesting for damaging the ECM by CAP treatment. If this could be achieved with CAP, it could decrease the intra-tumoral pressure and increase the perfusion in blood and lymphatic vessels. This could potentially boost the delivery of chemotherapeutics and infiltration of cells of the immune system (therefore, also favoring immunotherapy) to eliminate cancer cells. It has recently been observed that CAP treatment for wound healing increases tissue oxygenation and perfusion [119]. This finding could suggest that indeed CAP can weaken the ECM and allow better perfusion in blood and lymphatic vessels by decreasing intra-tumoral pressure.

### 5.3. Autophagic Cell Death

Autophagic cell death in PSCs, induced by PCCs, serves as a strategy to provide alternative nutrients to PCCs and has been related to a poor prognosis for PDAC patients [120]. Is it possible to avoid this autophagic cell death of PSCs by eliminating PSCs through other cell death pathways, e.g., apoptosis, necrosis and ICD induced by CAP? Or is it possible to inhibit the autophagic cell death of PSCs by CAP-derived RONS? It has been found that PSC activation can be inhibited by suppressing PSC autophagy through the activation of the PI3K/AKT/mTOR signalling pathway [121,122,123]. mTOR and the PI3K/AKT/mTOR pathways were indeed suggested to inhibit autophagy [124,125,126]. Even though a controversy exists concerning the effect of RONS on the activation of this pathway, it has been suggested that RONS can activate it, therefore inhibiting autophagy in pancreatic cancer [127,128].

### 5.4. Inhibiting Immunosuppression

Another possibility is looking into the immunosuppressive character of PDAC, which is also a result of the TME provided by PSCs. The stimulation of the immune system upon CAP treatment has already been proven by the observation of ICD in PCCs [42]. However, co-cultures of PCCs and PSCs have not been investigated yet. It is necessary to obtain information on these co-cultures as well, as the cross-talk of both cell types leads to the typical immunosuppressive TME of PDAC. The recruitment of T-regs and MDSCs is another key process that has not yet been investigated either, as these cells are important mediators in creating immunosuppression.

### 5.5. Hedgehog Signaling

Sonic hedgehog (Shh) has been observed to be secreted in *KRAS*-mutated PCCs and induces non-cell-autonomous signaling on PSCs [129]. PSC-secreted proteins such as ECM components, e.g., collagens, and MMPs, were upregulated upon Shh signaling. In this way, Shh contributes to the characteristic desmoplasia in PDAC and is suspected to have an influence on the activation of the PSCs [129,130]. A drug that inhibits the Hedgehog (Hh) pathway (IPI-926) was able to reduce the abundance of stroma with an enhanced intra-tumoral delivery of gemcitabine [131]. This study reveals the contribution of the Hh family to the desmoplasia and the importance of inhibiting this pathway. Failure in inhibiting the Shh signaling in clinical trials obligated to re-evaluate this strategy [88]. However, Ma et al. showed that sanguinarine can inhibit the Shh pathway, and induces oxidative damage [132]. Therefore, it would be interesting to investigate the influence of oxidative damage by RONS due to CAP treatment on the Shh pathway.

### 5.6. Combinational Strategies

Due to the complexity of this disease, the combination of different therapies that target different pathways emerges as a promising strategy to achieving more efficient therapeutic responses. The combination of CAP treatment with chemotherapeutics could be highly interesting due to a potential synergistic effect, as in Section 1. Currently used chemotherapeutics for PDAC are FOLFIRINOX or gemcitabine plus nab-paclitaxel [133]. FOLFIRINOX is a combination of oxaliplatin, fluorouracil (5-FU), irinotecan and leucovorin, and works on quickly dividing cells [77,134]. The mechanisms of actions have been clearly described in a review by Pereira et al. [135]: 5-FU is an anti-metabolite that inhibits the synthesis of thymine and DNA synthesis. Leucovorin is a metabolite that reduces the adverse toxicity effects of 5-FU and contributes to prolonging the survival rates. Irinotecan has cytotoxic effects via the inhibition on DNA topoisomerase I, which induces DNA damage and leads to cell death. Oxaliplatin can bind guanine and cytosine, creating cross-linked DNA, which also leads to the inhibition of DNA synthesis and its transcription. The mechanism of gemcitabine involves the inhibition of DNA synthesis by a so-called masked chain-termination, causing the inability of gemcitabine removal by DNA repair mechanisms [136]. Paclitaxel is based on the inhibition of the depolymerization of microtubules, which blocks the cell cycle and leads to cell death [137]. Nab-paclitaxel is albumin-bound paclitaxel and has an improved pharmacokinetics profile, which leads to a higher response to this chemotherapeutic [137]. As DNA damage is one of the many induced effects of CAP on cells, it is highly possible that a combination of these chemotherapeutics with CAP will enhance their cytotoxic effect on cancer cells. Using a direct treatment of CAP treatment would only affect the cancer and normal cells locally, reducing excessive side effects of the combination of both on normal cells.

Different methods have already been used to reduce PSC proliferation, activation and their ECM production and are described in a review by Pang et al. [138]. All-trans retinoic acid (ATRA), a compound that induces PSC quiescence and was found to reduce PSC proliferation, increases PSC apoptosis and decreases ECM production [139]. Scoparone can downregulate the TGF-β pathway which reduces PSC activation [140]. Metformin can inhibit the activation of PSCs and reduce tumor size in mice, and its combination with gemcitabine further enhances this effect [141,142]. These anti-stromal compounds and others could be combined with CAP treatment to evaluate a possible additive or synergistic effect.

### 5.7. Co-Cultures of PCCs and PSCs

Last but not least, while the mono-cultures of PCCs and PSCs provide valuable information on the effects of CAP on each population, it is highly necessary to investigate co-cultures of PCCs and PSCs (using both 3D in vitro and in vivo models), as they can more accurately mimic the complex interactions between PCCs and PSCs in the TME. The cross-talk of both cell types is a major contribution to the aggressiveness of the disease and should therefore be taken into account when exploring a new therapeutic approach for PDAC. This strategy has already been used for experimental models for pancreatic cancer desmoplasia and should also be considered for research on CAP treatment for PDAC [131].

## 6. Conclusions

CAP is a novel therapeutic strategy for cancer treatment and could potentially be used to treat hard-to-kill PDAC in combination with other therapeutic approaches, such as chemotherapy. Although research in this field is still in an early stage, this review highlights the potential of CAP treatment for PDAC, as demonstrated in the state-of-the-art section, but also shows that more research in this field is necessary. Since PSCs play an important role for PDAC in the creation of an immunosuppressive, inflammatory, and resilient TME to serve tumor progress and survival, it is important to evaluate the effect of CAP on PSCs to predict an accurate response to therapy of in vivo tumors. Currently, only two studies have investigated the effect of CAP on PSCs as mono-cultures, but no studies have been performed with co-cultures of PCCs and PSCs. This is a crucial point because the cross-talk between both cell types causes the aggressiveness of this disease. In addition, in this review we discussed the unknown effects of CAP treatment in PDAC, asking key questions that should be explored in the future of CAP therapy: PSC reprogramming possibilities, autophagic cell death, the role of the extracellular matrix, the recruitment of immunosuppressive cells and cytokines, and the combination of CAP with chemotherapeutics, to evaluate whether an enhanced or synergistic effect is observed.

## Figures and Tables

**Table 1:** List of markers for quiescent and activated PSCs. (x) = could be expressed, x = expressed, x(x) = expression could be increased, xx = increased expression.

Marker	Quiescent PSC	Activated PSC	References
Desmin	x	x(x)	[94,96,97,98,99]
Vimentin	x	x(x)	[94,95,97,99,100,101,102]
GFAP	x	x(x)	[94,96,97,98,99,103]
α-SMA		xx	[79,94,97,99,104,105]
FAP	(x)	x	[105,106]
E-cadherin	xx	x	[95,101,102]
N-cadherin	x	xx	[95,102]
Slug	x	x(x)	[95,102]

**Table 2:** Overview of the state-of-the-art on CAP treatment of PDAC.

PCC Cell Lines	PSC Cell Lines	Response Shown	Plasma Device	Treatment	Model	Reference
Murine 6606PDA		Surface temperature Cell death	Plasma jet	Direct	In vitro	[110]
Murine 6606PDA		Metabolic activity Cell proliferation Apoptosis	Plasma jet	Indirect Direct	In vitro In vivo	[6]
Mia PaCa-2		Combination gemcitabine and CAP on tumor growth	Plasma jet	Direct	In vitro In vivo	[43]
Mia PaCa-2 PANC-1		Metabolic activity Metastatic potential	Plasma jet	Direct	In vitro In ovo	[111]
BxPC-3 Mia PaCa-2 Capan-2 PANC-1		Apoptosis Cell viability Selectivity	Plasma jet	Indirect	In vitro In vivo	[112]
PANC-1		Cell viability Cell death	DBD	Indirect	In vitro	[113]
BxPC-3		Cell viability	Plasma jet	Indirect	In vitro	[53]
BxPC-3 Mia PaCa-2 Capan-2 PANC-1	hPSC128 hPSC21 RLT-PSC	Immunogenic cell death induction	Plasma jet	Indirect	In vitro	[42]
Mia PaCa-2 BxPC-3	hPSC128-SV	Cell viability	Plasma jet	Indirect	In vitro	[114]

## References

[1] Vogelstein B., Kinzler K.W. (2004). Cancer genes and the pathways they control. Nat. Med..

[2] Correia A.L., Bissell M.J. (2012). The tumor microenvironment is a dominant force in multidrug resistance. Drug Resist. Updat..

[3] Lin A., Gorbanev Y., De Backer J., Van Loenhout J., Van Boxem W., Lemiere F., Cos P., Dewilde S., Smits E., Bogaerts A. (2019). Non-Thermal Plasma as a Unique Delivery System of Short-Lived Reactive Oxygen and Nitrogen Species for Immunogenic Cell Death in Melanoma Cells. Adv. Sci. (Weinh).

[4] Guerrero-Preston R., Ogawa T., Uemura M., Shumulinsky G., Valle B.L., Pirini F., Ravi R., Sidransky D., Keidar M., Trink B. (2014). Cold atmospheric plasma treatment selectively targets head and neck squamous cell carcinoma cells. Int. J. Mol. Med..

[5] Fridman G., Shereshevsky A., Jost M.M., Brooks A.D., Fridman A., Gutsol A., Vasilets V., Friedman G. (2007). Floating Electrode Dielectric Barrier Discharge Plasma in Air Promoting Apoptotic Behavior in Melanoma Skin Cancer Cell Lines. Plasma Chem. Plasma Process..

[6] Liedtke K.R., Bekeschus S., Kaeding A., Hackbarth C., Kuehn J.P., Heidecke C.D., von Bernstorff W., von Woedtke T., Partecke L.I. (2017). Non-thermal plasma-treated solution demonstrates antitumor activity against pancreatic cancer cells in vitro and in vivo. Sci. Rep..

[7] Metelmann H.-R., Nedrelow D.S., Seebauer C., Schuster M., von Woedtke T., Weltmann K.-D., Kindler S., Metelmann P.H., Finkelstein S.E., Von Hoff D.D. (2015). Head and neck cancer treatment and physical plasma. Clin. Plasma Med..

[8] Metelmann H.-R., Seebauer C., Miller V., Fridman A., Bauer G., Graves D.B., Pouvesle J.-M., Rutkowski R., Schuster M., Bekeschus S. (2018). Clinical experience with cold plasma in the treatment of locally advanced head and neck cancer. Clin. Plasma Med..

[9] Bray F., Ferlay J., Soerjomataram I., Siegel R.L., Torre L.A., Jemal A. (2018). Global cancer statistics 2018: GLOBOCAN estimates of incidence and mortality worldwide for 36 cancers in 185 countries. CA Cancer J. Clin..

[10] Hasan S., Jacob R., Manne U., Paluri R. (2019). Advances in pancreatic cancer biomarkers. Oncol. Rev..

[11] Gouarderes S., Mingotaud A.-F., Vicendo P., Gibot L. (2020). Vascular and extracellular matrix remodeling by physical approaches to improve drug delivery at the tumor site. Exp. Opin. Drug Deliv..

[12] Moreau M., Orange N., Feuilloley M.G.J. (2008). Non-thermal plasma technologies: New tools for bio-decontamination. Biotechnol. Adv..

[13] Fridman A.A. (2012). Plasma Chemistry.

[14] Fridman A.A., Friedman G. (2013). Plasma Medicine.

[15] Dubuc A., Monsarrat P., Virard F., Merbahi N., Sarrette J.P., Laurencin-Dalicieux S., Cousty S. (2018). Use of cold-atmospheric plasma in oncology: A concise systematic review. Ther. Adv. Med. Oncol..

[16] Privat-Maldonado A., Schmidt A., Lin A., Weltmann K.D., Wende K., Bogaerts A., Bekeschus S. (2019). ROS from Physical Plasmas: Redox Chemistry for Biomedical Therapy. Oxid. Med. Cell. Longev..

[17] Yan D., Sherman J.H., Keidar M. (2016). Cold atmospheric plasma, a novel promising anti-cancer treatment modality. Oncotarget.

[18] Snoeckx R., Bogaerts A. (2017). Plasma technology—A novel solution for CO2 conversion?. Chem. Soc. Rev..

[19] Biscop E., Lin A., Boxem W.V., Loenhout J.V., Backer J., Deben C., Dewilde S., Smits E., Bogaerts A.A. (2019). Influence of Cell Type and Culture Medium on Determining Cancer Selectivity of Cold Atmospheric Plasma Treatment. Cancers (Basel).

[20] Bekeschus S., Schmidt A., Weltmann K.-D., von Woedtke T. (2016). The plasma jet kINPen—A powerful tool for wound healing. Clin. Plasma Med..

[21] Hoffmann C., Berganza C., Zhang J. (2013). Cold Atmospheric Plasma: Methods of production and application in dentistry and oncology. Med. Gas. Res..

[22] Yarmolenko P.S., Moon E.J., Landon C., Manzoor A., Hochman D.W., Viglianti B.L., Dewhirst M.W. (2011). Thresholds for thermal damage to normal tissues: An update. Int. J. Hyperth..

[23] Fridman G., Friedman G., Gutsol A., Shekhter A.B., Vasilets V.N., Fridman A. (2008). Applied Plasma Medicine. Plasma Process. Polym..

[24] Rahal A., Kumar A., Singh V., Yadav B., Tiwari R., Chakraborty S., Dhama K. (2014). Oxidative stress, prooxidants, and antioxidants: The interplay. Biomed. Res. Int..

[25] Tovmasyan A., Maia C.G.C., Weitner T., Carballal S., Sampaio R.S., Lieb D., Ghazaryan R., Ivanovic-Burmazovic I., Ferrer-Sueta G., Radi R. (2015). A comprehensive evaluation of catalase-like activity of different classes of redox-active therapeutics. Free Radic. Biol. Med..

[26] Tanaka H., Mizuno M., Ishikawa K., Toyokuni S., Kajiyama H., Kikkawa F., Hori M. (2018). Molecular mechanisms of non-thermal plasma-induced effects in cancer cells. Biol. Chem..

[27] Bauer G., Graves D.B. (2016). Mechanisms of Selective Antitumor Action of Cold Atmospheric Plasma-Derived Reactive Oxygen and Nitrogen Species. Plasma Process. Polym..

[28] Finkel T. (2011). Signal transduction by reactive oxygen species. J. Cell Biol..

[29] Yan D., Xiao H., Zhu W., Nourmohammadi N., Zhang L.G., Bian K., Keidar M. (2017). The role of aquaporins in the anti-glioblastoma capacity of the cold plasma-stimulated medium. J. Phys. D Appl. Phys..

[30] Vilema-Enriquez G., Arroyo A., Grijalva M., Amador-Zafra R.I., Camacho J. (2016). Molecular and Cellular Effects of Hydrogen Peroxide on Human Lung Cancer Cells: Potential Therapeutic Implications. Oxid. Med. Cell Longev..

[31] Tanaka H., Mizuno M., Ishikawa K., Nakamura K., Kajiyama H., Kano H., Kikkawa F., Hori M. (2011). Plasma-Activated Medium Selectively Kills Glioblastoma Brain Tumor Cells by Down-Regulating a Survival Signaling Molecule, AKT Kinase. Plasma Med..

[32] Ebrahimi S., Hosseini M., Shahidsales S., Maftouh M., Ferns G.A., Ghayour-Mobarhan M., Hassanian S.M., Avan A. (2017). Targeting the Akt/PI3K Signaling Pathway as a Potential Therapeutic Strategy for the Treatment of Pancreatic Cancer. Curr. Med. Chem..

[33] Cheng H., Xu J., Li X., Liu D., Lu X. (2020). On the dose of plasma medicine: Equivalent total oxidation potential (ETOP). Phys. Plasmas.

[34] Vandamme M., Robert E., Lerondel S., Sarron V., Ries D., Dozias S., Sobilo J., Gosset D., Kieda C., Legrain B. (2012). ROS implication in a new antitumor strategy based on non-thermal plasma. Int. J. Cancer.

[35] Weltmann K.D., von Woedtke T. (2016). Plasma medicine—Current state of research and medical application. Plasma Phys. Control. Fusion.

[36] Calabrese E.J., Baldwin L.A. (2002). Defining hormesis. Hum. Exp. Toxicol..

[37] Leutner S., Eckert A., Muller W.E. (2001). ROS generation, lipid peroxidation and antioxidant enzyme activities in the aging brain. J. Neural Transm. (Vienna).

[38] Hirst A.M., Frame F.M., Maitland N.J., O’Connell D. (2014). Low Temperature Plasma Causes Double-Strand Break DNA Damage in Primary Epithelial Cells Cultured from a Human Prostate Tumour. IEEE Trans. Plasma Sci. IEEE Nucl. Plasma Sci. Soc..

[39] Weiss M., Gumbel D., Hanschmann E.M., Mandelkow R., Gelbrich N., Zimmermann U., Walther R., Ekkernkamp A., Sckell A., Kramer A. (2015). Cold Atmospheric Plasma Treatment Induces Anti-Proliferative Effects in Prostate Cancer Cells by Redox and Apoptotic Signaling Pathways. PLoS ONE.

[40] Keidar M., Yan D., Beilis I.I., Trink B., Sherman J.H. (2018). Plasmas for Treating Cancer: Opportunities for Adaptive and Self-Adaptive Approaches. Trends Biotechnol..

[41] Ma Y., Ha C.S., Hwang S.W., Lee H.J., Kim G.C., Lee K.W., Song K. (2014). Non-thermal atmospheric pressure plasma preferentially induces apoptosis in p53-mutated cancer cells by activating ROS stress-response pathways. PLoS ONE.

[42] Van Loenhout J., Flieswasser T., Freire Boullosa L., De Waele J., Van Audenaerde J., Marcq E., Jacobs J., Lin A., Lion E., Dewitte H. (2019). Cold Atmospheric Plasma-Treated PBS Eliminates Immunosuppressive Pancreatic Stellate Cells and Induces Immunogenic Cell Death of Pancreatic Cancer Cells. Cancers (Basel).

[43] Brulle L., Vandamme M., Ries D., Martel E., Robert E., Lerondel S., Trichet V., Richard S., Pouvesle J.M., Le Pape A. (2012). Effects of a non thermal plasma treatment alone or in combination with gemcitabine in a MIA PaCa2-luc orthotopic pancreatic carcinoma model. PLoS ONE.

[44] Lin L., Wang L., Liu Y., Xu C., Tu Y., Zhou J. (2018). Nonthermal plasma inhibits tumor growth and proliferation and enhances the sensitivity to radiation in vitro and in vivo. Oncol. Rep..

[45] Panngom K., Baik K.Y., Nam M.K., Han J.H., Rhim H., Choi E.H. (2013). Preferential killing of human lung cancer cell lines with mitochondrial dysfunction by nonthermal dielectric barrier discharge plasma. Cell Death Dis..

[46] Keidar M., Walk R., Shashurin A., Srinivasan P., Sandler A., Dasgupta S., Ravi R., Guerrero-Preston R., Trink B. (2011). Cold plasma selectivity and the possibility of a paradigm shift in cancer therapy. Br. J. Cancer.

[47] Schuster M., Seebauer C., Rutkowski R., Hauschild A., Podmelle F., Metelmann C., Metelmann B., von Woedtke T., Hasse S., Weltmann K.D. (2016). Visible tumor surface response to physical plasma and apoptotic cell kill in head and neck cancer. J. Craniomaxillofac. Surg..

[48] Wang M., Holmes B., Cheng X., Zhu W., Keidar M., Zhang L.G. (2013). Cold atmospheric plasma for selectively ablating metastatic breast cancer cells. PLoS ONE.

[49] Zhu W., Lee S.J., Castro N.J., Yan D., Keidar M., Zhang L.G. (2016). Synergistic Effect of Cold Atmospheric Plasma and Drug Loaded Core-shell Nanoparticles on Inhibiting Breast Cancer Cell Growth. Sci. Rep..

[50] Sensenig R., Kalghatgi S., Cerchar E., Fridman G., Shereshevsky A., Torabi B., Arjunan K.P., Podolsky E., Fridman A., Friedman G. (2011). Non-thermal plasma induces apoptosis in melanoma cells via production of intracellular reactive oxygen species. Ann. Biomed. Eng..

[51] Plewa J.-M., Yousfi M., Frongia C., Eichwald O., Ducommun B., Merbahi N., Lobjois V. (2014). Low-temperature plasma-induced antiproliferative effects on multi-cellular tumor spheroids. New J. Phys..

[52] Kim C.H., Kwon S., Bahn J.H., Lee K., Jun S.I., Rack P.D., Baek S.J. (2010). Effects of atmospheric nonthermal plasma on invasion of colorectal cancer cells. Appl. Phys. Lett..

[53] Chen Z., Lin L., Gjika E., Cheng X., Canady J., Keidar M. (2018). Selective Treatment of Pancreatic Cancer Cells by Plasma-Activated Saline Solutions. IEEE Trans. Radiat. Plasma Med. Sci..

[54] Miller V., Lin A., Fridman A. (2016). Why Target Immune Cells for Plasma Treatment of Cancer. Plasma Chem. Plasma Process..

[55] Zucker S.N., Zirnheld J., Bagati A., DiSanto T.M., Des Soye B., Wawrzyniak J.A., Etemadi K., Nikiforov M., Berezney R. (2012). Preferential induction of apoptotic cell death in melanoma cells as compared with normal keratinocytes using a non-thermal plasma torch. Cancer Biol. Ther..

[56] Kaushik N., Kumar N., Kim C.H., Kaushik N.K., Choi E.H. (2014). Dielectric Barrier Discharge Plasma Efficiently Delivers an Apoptotic Response in Human Monocytic Lymphoma. Plasma Process. Polym..

[57] Utsumi F., Kajiyama H., Nakamura K., Tanaka H., Hori M., Kikkawa F. (2014). Selective cytotoxicity of indirect nonequilibrium atmospheric pressure plasma against ovarian clear-cell carcinoma. Springerplus.

[58] Kim S.J., Chung T.H. (2016). Cold atmospheric plasma jet-generated RONS and their selective effects on normal and carcinoma cells. Sci. Rep..

[59] Hirst A.M., Frame F.M., Arya M., Maitland N.J., O’Connell D. (2016). Low temperature plasmas as emerging cancer therapeutics: The state of play and thoughts for the future. Tumour Biol..

[60] Keidar M. (2018). A prospectus on innovations in the plasma treatment of cancer. Phys. Plasmas.

[61] Hubenak J.R., Zhang Q., Branch C.D., Kronowitz S.J. (2014). Mechanisms of injury to normal tissue after radiotherapy: A review. Plast. Reconstr. Surg..

[62] Yan D., Talbot A., Nourmohammadi N., Sherman J.H., Cheng X., Keidar M. (2015). Toward understanding the selective anticancer capacity of cold atmospheric plasma--a model based on aquaporins (Review). Biointerphases.

[63] Yusupov M., Razzokov J., Cordeiro R.M., Bogaerts A. (2019). Transport of Reactive Oxygen and Nitrogen Species across Aquaporin: A Molecular Level Picture. Oxid. Med. Cell Longev..

[64] Yusupov M., Yan D., Cordeiro R.M., Bogaerts A. (2018). Atomic scale simulation of H2O2permeation through aquaporin: Toward the understanding of plasma cancer treatment. J. Phys. D Appl. Phys..

[65] Van der Paal J., Verheyen C., Neyts E.C., Bogaerts A. (2017). Hampering Effect of Cholesterol on the Permeation of Reactive Oxygen Species through Phospholipids Bilayer: Possible Explanation for Plasma Cancer Selectivity. Sci. Rep..

[66] Van der Paal J., Neyts E.C., Verlackt C.C.W., Bogaerts A. (2016). Effect of lipid peroxidation on membrane permeability of cancer and normal cells subjected to oxidative stress. Chem. Sci..

[67] Orth M., Metzger P., Gerum S., Mayerle J., Schneider G., Belka C., Schnurr M., Lauber K. (2019). Pancreatic ductal adenocarcinoma: Biological hallmarks, current status, and future perspectives of combined modality treatment approaches. Radiat. Oncol..

[68] Siegel R., Naishadham D., Jemal A. (2013). Cancer statistics, 2013. CA Cancer J. Clin..

[69] Siegel R.L., Miller K.D., Jemal A. (2017). Cancer Statistics, 2017. CA Cancer J. Clin..

[70] Mekapogu A.R., Pothula S.P., Pirola R.C., Wilson J.S., Apte M.V. (2019). Multifunctional role of pancreatic stellate cells in pancreatic cancer. Ann. Pancreat. Cancer.

[71] Wang L., Xie D., Wei D. (2019). Pancreatic Acinar-to-Ductal Metaplasia and Pancreatic Cancer. Methods Mol. Biol..

[72] Apte M.V., Xu Z., Pothula S., Goldstein D., Pirola R.C., Wilson J.S. (2015). Pancreatic cancer: The microenvironment needs attention too!. Pancreatology.

[73] McGuigan A., Kelly P., Turkington R.C., Jones C., Coleman H.G., McCain R.S. (2018). Pancreatic cancer: A review of clinical diagnosis, epidemiology, treatment and outcomes. World J. Gastroenterol..

[74] Fogel E.L., Shahda S., Sandrasegaran K., DeWitt J., Easler J.J., Agarwal D.M., Eagleson M., Zyromski N.J., House M.G., Ellsworth S. (2017). A Multidisciplinary Approach to Pancreas Cancer in 2016: A Review. Am. J. Gastroenterol..

[75] Hackert T. (2018). Surgery for Pancreatic Cancer after neoadjuvant treatment. Ann. Gastroenterol. Surg..

[76] Williet N., Saint A., Pointet A.L., Tougeron D., Pernot S., Pozet A., Bechade D., Trouilloud I., Lourenco N., Hautefeuille V. (2019). Folfirinox versus gemcitabine/nab-paclitaxel as first-line therapy in patients with metastatic pancreatic cancer: A comparative propensity score study. Therap. Adv. Gastroenterol..

[77] Conroy T., Desseigne F., Ychou M., Bouche O., Guimbaud R., Becouarn Y., Adenis A., Raoul J.L., Gourgou-Bourgade S., de la Fouchardiere C. (2011). FOLFIRINOX versus gemcitabine for metastatic pancreatic cancer. N. Engl. J. Med..

[78] Von Hoff D.D., Ervin T., Arena F.P., Chiorean E.G., Infante J., Moore M., Seay T., Tjulandin S.A., Ma W.W., Saleh M.N. (2013). Increased survival in pancreatic cancer with nab-paclitaxel plus gemcitabine. N. Engl. J. Med..

[79] Erkan M., Adler G., Apte M.V., Bachem M.G., Buchholz M., Detlefsen S., Esposito I., Friess H., Gress T.M., Habisch H.J. (2012). StellaTUM: Current consensus and discussion on pancreatic stellate cell research. Gut.

[80] Brunner T.B., Scott-Brown M. (2010). The role of radiotherapy in multimodal treatment of pancreatic carcinoma. Radiat. Oncol..

[81] Couzin-Frankel J. (2013). Breakthrough of the year 2013. Cancer immunotherapy. Science.

[82] Schizas D., Charalampakis N., Kole C., Economopoulou P., Koustas E., Gkotsis E., Ziogas D., Psyrri A., Karamouzis M.V. (2020). Immunotherapy for pancreatic cancer: A 2020 update. Cancer Treat. Rev..

[83] Brahmer J.R., Tykodi S.S., Chow L.Q., Hwu W.J., Topalian S.L., Hwu P., Drake C.G., Camacho L.H., Kauh J., Odunsi K. (2012). Safety and activity of anti-PD-L1 antibody in patients with advanced cancer. N. Engl. J. Med..

[84] Torphy R.J., Zhu Y., Schulick R.D. (2018). Immunotherapy for pancreatic cancer: Barriers and breakthroughs. Ann. Gastroenterol. Surg..

[85] Xu J.W., Wang L., Cheng Y.G., Zhang G.Y., Hu S.Y., Zhou B., Zhan H.X. (2018). Immunotherapy for pancreatic cancer: A long and hopeful journey. Cancer Lett..

[86] Rosenberg S.A. (2014). Decade in review-cancer immunotherapy: Entering the mainstream of cancer treatment. Nat. Rev. Clin. Oncol..

[87] Singh R.R., O’Reilly E.M. (2020). New Treatment Strategies for Metastatic Pancreatic Ductal Adenocarcinoma. Drugs.

[88] Schnittert J., Bansal R., Prakash J. (2019). Targeting Pancreatic Stellate Cells in Cancer. Trends Cancer.

[89] Mace T.A., Bloomston M., Lesinski G.B. (2013). Pancreatic cancer-associated stellate cells: A viable target for reducing immunosuppression in the tumor microenvironment. Oncoimmunology.

[90] Ferdek P.E., Jakubowska M.A. (2017). Biology of pancreatic stellate cells-more than just pancreatic cancer. Pflug. Arch.-Eur. J. Physiol..

[91] Graeber T.G., Osmanian C., Jacks T., Housman D.E., Koch C.J., Lowe S.W., Giaccia A.J. (1996). Hypoxia-mediated selection of cells with diminished apoptotic potential in solid tumours. Nature.

[92] Sousa C.M., Biancur D.E., Wang X., Halbrook C.J., Sherman M.H., Zhang L., Kremer D., Hwang R.F., Witkiewicz A.K., Ying H. (2016). Pancreatic stellate cells support tumour metabolism through autophagic alanine secretion. Nature.

[93] Masamune A., Kikuta K., Watanabe T., Satoh K., Hirota M., Shimosegawa T. (2008). Hypoxia stimulates pancreatic stellate cells to induce fibrosis and angiogenesis in pancreatic cancer. Am. J. Physiol. Gastrointest. Liver Physiol..

[94] Apte M.V., Pirola R.C., Wilson J.S. (2012). Pancreatic stellate cells: A starring role in normal and diseased pancreas. Front. Physiol..

[95] Tian L., Lu Z.P., Cai B.B., Zhao L.T., Qian D., Xu Q.C., Wu P.F., Zhu Y., Zhang J.J., Du Q. (2016). Activation of pancreatic stellate cells involves an EMT-like process. Int. J. Oncol..

[96] Pothula S.P., Xu Z., Goldstein D., Pirola R.C., Wilson J.S., Apte M.V. (2016). Key role of pancreatic stellate cells in pancreatic cancer. Cancer Lett..

[97] Jesnowski R., Furst D., Ringel J., Chen Y., Schrodel A., Kleeff J., Kolb A., Schareck W.D., Lohr M. (2005). Immortalization of pancreatic stellate cells as an in vitro model of pancreatic fibrosis: Deactivation is induced by matrigel and N-acetylcysteine. Lab. Investig..

[98] Apte M.V., Phillips P.A., Fahmy R.G., Darby S.J., Rodgers S.C., McCaughan G.W., Korsten M.A., Pirola R.C., Naidoo D., Wilson J.S. (2000). Does alcohol directly stimulate pancreatic fibrogenesis? Studies with rat pancreatic stellate cells. Gastroenterology.

[99] Bachem M.G., Zhou Z., Zhou S., Siech M. (2006). Role of stellate cells in pancreatic fibrogenesis associated with acute and chronic pancreatitis. J. Gastroenterol. Hepatol..

[100] Masamune A., Satoh A., Watanabe T., Kikuta K., Satoh M., Suzuki N., Satoh K., Shimosegawa T. (2010). Effects of ethanol and its metabolites on human pancreatic stellate cells. Dig. Dis. Sci..

[101] Kikuta K., Masamune A., Watanabe T., Ariga H., Itoh H., Hamada S., Satoh K., Egawa S., Unno M., Shimosegawa T. (2010). Pancreatic stellate cells promote epithelial-mesenchymal transition in pancreatic cancer cells. Biochem. Biophys. Res. Commun..

[102] Wu Y.S., Chung I., Wong W.F., Masamune A., Sim M.S., Looi C.Y. (2017). Paracrine IL-6 signaling mediates the effects of pancreatic stellate cells on epithelial-mesenchymal transition via Stat3/Nrf2 pathway in pancreatic cancer cells. Biochim. Biophys. Acta Gen. Subj..

[103] Han S., Delitto D., Zhang D., Sorenson H.L., Sarosi G.A., Thomas R.M., Behrns K.E., Wallet S.M., Trevino J.G., Hughes S.J. (2015). Primary outgrowth cultures are a reliable source of human pancreatic stellate cells. Lab. Investig..

[104] Omary M.B., Lugea A., Lowe A.W., Pandol S.J. (2007). The pancreatic stellate cell: A star on the rise in pancreatic diseases. J. Clin. Investig..

[105] Lunardi S., Muschel R.J., Brunner T.B. (2014). The stromal compartments in pancreatic cancer: Are there any therapeutic targets?. Cancer Lett..

[106] Lee H.O., Mullins S.R., Franco-Barraza J., Valianou M., Cukierman E., Cheng J.D. (2011). FAP-overexpressing fibroblasts produce an extracellular matrix that enhances invasive velocity and directionality of pancreatic cancer cells. BMC Cancer.

[107] Cabrera M.C., Tilahun E., Nakles R., Diaz-Cruz E.S., Charabaty A., Suy S., Jackson P., Ley L., Slack R., Jha R. (2014). Human Pancreatic Cancer-Associated Stellate Cells Remain Activated after in vivo Chemoradiation. Front. Oncol..

[108] Birbrair A. (2020). Tumor Microenvironment.

[109] Sun Q., Zhang B., Hu Q., Qin Y., Xu W., Liu W., Yu X., Xu J. (2018). The impact of cancer-associated fibroblasts on major hallmarks of pancreatic cancer. Theranostics.

[110] Partecke L.I., Evert K., Haugk J., Doering F., Normann L., Diedrich S., Weiss F.U., Evert M., Huebner N.O., Guenther C. (2012). Tissue tolerable plasma (TTP) induces apoptosis in pancreatic cancer cells in vitro and in vivo. BMC Cancer.

[111] Bekeschus S., Freund E., Spadola C., Privat-Maldonado A., Hackbarth C., Bogaerts A., Schmidt A., Wende K., Weltmann K.D., von Woedtke T. (2019). Risk Assessment of kINPen Plasma Treatment of Four Human Pancreatic Cancer Cell Lines with Respect to Metastasis. Cancers (Basel).

[112] Hattori N., Yamada S., Torii K., Takeda S., Nakamura K., Tanaka H., Kajiyama H., Kanda M., Fujii T., Nakayama G. (2015). Effectiveness of plasma treatment on pancreatic cancer cells. Int. J. Oncol..

[113] Azzariti A., Iacobazzi R.M., Di Fonte R., Porcelli L., Gristina R., Favia P., Fracassi F., Trizio I., Silvestris N., Guida G. (2019). Plasma-activated medium triggers cell death and the presentation of immune activating danger signals in melanoma and pancreatic cancer cells. Sci. Rep..

[114] Kumar N., Attri P., Dewilde S., Bogaerts A. (2018). Inactivation of human pancreatic ductal adenocarcinoma with atmospheric plasma treated media and water: A comparative study. J. Phys. D Appl. Phys..

[115] Zhou J., Wang G., Chen Y., Wang H., Hua Y., Cai Z. (2019). Immunogenic cell death in cancer therapy: Present and emerging inducers. J. Cell Mol. Med..

[116] Saison-Ridinger M., DelGiorno K.E., Zhang T., Kraus A., French R., Jaquish D., Tsui C., Erikson G., Spike B.T., Shokhirev M.N. (2017). Reprogramming pancreatic stellate cells via p53 activation: A putative target for pancreatic cancer therapy. PLoS ONE.

[117] Harris S.L., Levine A.J. (2005). The p53 pathway: Positive and negative feedback loops. Oncogene.

[118] Eble J.A., de Rezende F.F. (2014). Redox-relevant aspects of the extracellular matrix and its cellular contacts via integrins. Antioxid. Redox Signal..

[119] Schmidt A., Nießner F., Woedtke T.V., Bekeschus S. (2020). Hyperspectral Imaging of Wounds Reveals Augmented Tissue Oxygenation Following Cold Physical Plasma Treatment in Vivo. IEEE Trans. Radiat. Plasma Med. Sci..

[120] Endo S., Nakata K., Ohuchida K., Takesue S., Nakayama H., Abe T., Koikawa K., Okumura T., Sada M., Horioka K. (2017). Autophagy Is Required for Activation of Pancreatic Stellate Cells, Associated With Pancreatic Cancer Progression and Promotes Growth of Pancreatic Tumors in Mice. Gastroenterology.

[121] Xue R., Yang J., Wu J., Meng Q., Hao J. (2017). Coenzyme Q10 inhibits the activation of pancreatic stellate cells through PI3K/AKT/mTOR signaling pathway. Oncotarget.

[122] Xue R., Wang J., Yang L., Liu X., Gao Y., Pang Y., Wang Y., Hao J. (2019). Coenzyme Q10 Ameliorates Pancreatic Fibrosis via the ROS-Triggered mTOR Signaling Pathway. Oxid. Med. Cell. Longev..

[123] Cui L.H., Li C.X., Zhuo Y.Z., Yang L., Cui N.Q., Zhang S.K. (2019). Saikosaponin d ameliorates pancreatic fibrosis by inhibiting autophagy of pancreatic stellate cells via PI3K/Akt/mTOR pathway. Chem. Biol. Interact..

[124] Yu L., McPhee C.K., Zheng L., Mardones G.A., Rong Y., Peng J., Mi N., Zhao Y., Liu Z., Wan F. (2010). Termination of autophagy and reformation of lysosomes regulated by mTOR. Nature.

[125] Racanelli A.C., Kikkers S.A., Choi A.M.K., Cloonan S.M. (2018). Autophagy and inflammation in chronic respiratory disease. Autophagy.

[126] Mathew R., Karantza-Wadsworth V., White E. (2007). Role of autophagy in cancer. Nat. Rev. Cancer.

[127] Fiorini C., Cordani M., Gotte G., Picone D., Donadelli M. (2015). Onconase induces autophagy sensitizing pancreatic cancer cells to gemcitabine and activates Akt/mTOR pathway in a ROS-dependent manner. Biochim. Biophys. Acta.

[128] Zhang L., Li J., Zong L., Chen X., Chen K., Jiang Z., Nan L., Li X., Li W., Shan T. (2016). Reactive Oxygen Species and Targeted Therapy for Pancreatic Cancer. Oxid. Med. Cell. Longev..

[129] Tape C.J., Ling S., Dimitriadi M., McMahon K.M., Worboys J.D., Leong H.S., Norrie I.C., Miller C.J., Poulogiannis G., Lauffenburger D.A. (2016). Oncogenic KRAS Regulates Tumor Cell Signaling via Stromal Reciprocation. Cell.

[130] Yu Y., Cheng L., Yan B., Zhou C., Qian W., Xiao Y., Qin T., Cao J., Han L., Ma Q. (2018). Overexpression of Gremlin 1 by sonic hedgehog signaling promotes pancreatic cancer progression. Int. J. Oncol..

[131] Suklabaidya S., Dash P., Das B., Suresh V., Sasmal P.K., Senapati S. (2018). Experimental models of pancreatic cancer desmoplasia. Lab. Investig..

[132] Ma Y., Yu W., Shrivastava A., Alemi F., Lankachandra K., Srivastava R.K., Shankar S. (2017). Sanguinarine inhibits pancreatic cancer stem cell characteristics by inducing oxidative stress and suppressing sonic hedgehog-Gli-Nanog pathway. Carcinogenesis.

[133] Springfeld C., Jager D., Buchler M.W., Strobel O., Hackert T., Palmer D.H., Neoptolemos J.P. (2019). Chemotherapy for pancreatic cancer. Presse Med..

[134] FOLFIRINOX. https://www.cancerresearchuk.org/about-cancer/cancer-in-general/treatment/cancer-drugs/drugs/folfirinox.

[135] Pereira N.P., Corrêa J.R. (2018). Pancreatic cancer: Treatment approaches and trends. J. Cancer Metastasis Treat..

[136] de Sousa Cavalcante L., Monteiro G. (2014). Gemcitabine: Metabolism and molecular mechanisms of action, sensitivity and chemoresistance in pancreatic cancer. Eur. J. Pharmacol..

[137] Yardley D.A. (2013). nab-Paclitaxel mechanisms of action and delivery. J. Control. Release.

[138] Pang T.C.Y., Wilson J.S., Apte M.V. (2017). Pancreatic stellate cells: What’s new?. Curr. Opin. Gastroenterol..

[139] Robinson B.K., Cortes E., Rice A.J., Sarper M., del Río Hernández A. (2016). Quantitative analysis of 3D extracellular matrix remodelling by pancreatic stellate cells. Biol. Open.

[140] Xu M., Cai J., Wei H., Zhou M., Xu P., Huang H., Peng W., Du F., Gong A., Zhang Y. (2016). Scoparone Protects Against Pancreatic Fibrosis via TGF-beta/Smad Signaling in Rats. Cell Physiol. Biochem..

[141] Duan W., Chen K., Jiang Z., Chen X., Sun L., Li J., Lei J., Xu Q., Ma J., Li X. (2017). Desmoplasia suppression by metformin-mediated AMPK activation inhibits pancreatic cancer progression. Cancer Lett..

[142] Zechner D., Burtin F., Albert A.C., Zhang X., Kumstel S., Schonrogge M., Graffunder J., Shih H.Y., Muller S., Radecke T. (2016). Intratumoral heterogeneity of the therapeutical response to gemcitabine and metformin. Oncotarget.

